# A systematic review of agomelatine-induced liver injury

**DOI:** 10.1186/s40303-015-0011-7

**Published:** 2015-04-21

**Authors:** Silka Dawn Freiesleben, Karolina Furczyk

**Affiliations:** Department of Psychiatry, University of Rostock, Gehlsheimerstraße 20, 18147 Rostock, Germany

**Keywords:** Adverse event, Antidepressant, Clinical recommendations, Depression, Hepatotoxicity, Melatonin analogue

## Abstract

Agomelatine is an antidepressant with a unique mechanism of action. Since its marketing in 2009, concerns have been raised regarding its potential to induce liver injury. The authors therefore address the need to comprehensively evaluate the potential risk posed by agomelatine of inducing liver injury by reviewing data from published and unpublished clinical trials in both the pre- and postmarketing settings, as well as data from non-interventional studies, pharmacovigilance database reviews and one case report. Recommendations for clinicians are also provided.

In this review, agomelatine was found to be associated with higher rates of liver injury than both placebo and the four active comparator antidepressants used in the clinical trials for agomelatine, with rates as high as 4.6% for agomelatine compared to 2.1% for placebo, 1.4% for escitalopram, 0.6% for paroxetine, 0.4% for fluoxetine, and 0% for sertraline. The review also provides evidence for the existence of a positive relationship between agomelatine dose and liver injury. Furthermore, rates of liver injury were found to be lower in non-interventional studies. Findings from pharmacovigilance database reviews and one case report also highlight the risk of agomelatine-induced liver injury.

As agomelatine does pose a risk of liver injury, clinicians must carefully monitor liver function throughout treatment. However, agomelatine’s unique mechanism of action and favourable safety profile render it a valuable treatment option.

A quantitative analysis of agomelatine-induced liver injury is lacking in the literature and would be welcomed.

## Introduction

Synthesized for the first time in 1992 [1], agomelatine has been licensed for the treatment of Major Depressive Disorder (MDD) in adults since 2009 in the European Union [2] and since 2010 in Australia [3].

Its short-term efficacy and tolerability have recently been systematically examined by Taylor et al. [4] using data from both published and unpublished clinical trials. In short, agomelatine was found to be more effective than placebo, and no more or less effective than other antidepressants (AD) in reducing acute symptoms of depression. Agomelatine was furthermore not found to be more effective than placebo or other ADs in achieving remission from depression. In a similar systematic review by Guaiana and colleagues [5] comparing agomelatine solely to other ADs, agomelatine was found to be as effective as other ADs in reducing acute symptoms of depression and in achieving remission.

Prior to, and after the marketing of agomelatine, concerns have been raised regarding its potential to induce liver injury, where liver injury, or hepatotoxicity, can be defined as injury to the liver that is associated with clinically significant elevations in liver serum transaminase titers of >3X upper limit of normal (ULN; [6,7]). Indeed, findings from premarketing trials have been pooled and list increases in liver serum transaminase titers of >3X ULN as a common side effect (seen in one in every 10-100 patient) [2,8]. Cases of serious hepatic reactions such as cytolytic hepatitis have also been reported to have occurred at a rate of one in every 1000-10 000 persons, rendering such occurrences as rare side-effects [2,8]. Liver enzyme elevations of >10X ULN have also occurred, with no exact data available [8]. Since its marketing, cases of serious hepatic reactions including hepatitis and jaundice, as well as liver enzyme elevations of >10X ULN have been reported to occur at a rate of one in every 1000-10 000 persons, rendering these occurrences as rare side effects [2,9]. To date, there have been six reported cases worldwide of hepatic failure resulting in death or liver transplantation in people with hepatic risk factors [10]. The manufacturer of agomelatine has responded to these concerns by emitting safety information letters and liver monitoring guidance protocols for the healthcare professionals prescribing agomelatine, the latest update being released in October 2013 [10].

As all ADs have the potential to induce liver injury even at therapeutic doses [7], and the most common reason for a drug to be rejected for application or withdrawn from the market in the United States being drug-induced liver injury (DILI; [11]), with similar guidelines applied by the European Medicines Agency [12], there is a need to critically evaluate and review the risk of DILI following the administration of agomelatine in the treatment of MDD in adults. Therefore, the following article will review the risk posed by agomelatine of inducing liver injury, and will attempt to determine if its benefits outweigh its’ risk of hepatotoxicity.

### Literature search

A PubMed search of full-text articles written in the English language using the search terms “agomelatine”, “agomelatine hepatotoxicity”, and “agomelatine liver” was conducted. Clinical trials, non-interventional studies, pharmacovigilance database reviews and case reports were identified. A total of 15 clinical trials were selected as blood samples or liver function tests were performed as part of their study design and as the results of these tests were reported. In addition, the unpublished data from the premarketing clinical trials of agomelatine with available results were reviewed from the European Medicines Agency [8] and the Novartis Clinical Trial Results Database [13]. We additionally consulted the ADFD Wissen website to obtain a complete list of all clinical trials sponsored by Servier® [14]. This generated a total of six additional articles that were selected. Three non-interventional studies, two pharmacovigilance database reviews and one case report were furthermore selected using these search terms.

To help contextualize our review, review articles with full-text availability were searched using the search terms “agomelatine liver” and “agomelatine hepatotoxicity”. This generated a total of 14 articles, of which two were selected for their relevance. Likewise, to obtain information on the AD efficacy of agomelatine, a search of systematic reviews with full-text availability was conducted using the terms “agomelatine antidepressant efficacy”. A total of 15 articles were generated, with five articles being selected for their relevance. Of these five review articles, we also consulted all relevant articles in their reference lists.

Lastly, Servier Laboratories were contacted to obtain additional unpublished data.

As no experimental research was conducted to create this review, no ethical approval has been applied for.

### Pharmacology of agomelatine

#### Mechanism of action

Circadian abnormalities are a central symptom in MDD pathology [15] (Li et al., 2013). In this sense, agomelatine is a unique ADs as it is a melatonin analogue that acts both as a melatonin-receptor agonist at MT_1_ and MT_2_ receptors [16] and a selective serotonin-receptor antagonist at 5-HT_2C_ receptors [17]. This unique mechanism of action confers agomelatine with the ability to regulate disrupted circadian rhythms by specifically: (1) inducing a phase advance of sleep, (2) reducing core body temperature, and by (3) positively phase shifting melatonin onset [2]. In an open-label EEG study with MDD patients, agomelatine has further been shown to increase slow-wave sleep duration and sleep efficiency [18]. Moreover, its antagonistic effects at 5-HT_2C_ receptors result in an increased extracellular release of noradrenaline and dopamine in the frontal cortex [17], endowing agomelatine with a more conventional AD profile as it affects mood control. Lastly, agomelatine has further been shown to have anxiolytic properties [19].

#### Absorption and distribution

Agomelatine is rapidly and almost completely absorbed after oral ingestion. After administration of 25-50 mg, maximum blood concentration is achieved between 45 and 90 minutes [8], and is absorbed at a rate of ≥ 80%. Intra- and inter-individual variability in bioavailability is considerable, and has been estimated to be higher in women compared to men, and higher in elderly compared to younger individuals. Therefore, sex and age are factors that have been suggested to affect the metabolic enzyme activity of agomelatine and hepatic blood flow [8].

#### Metabolism and biotransformation

90% of agomelatine is metabolised through the liver by the hepatic cytochrome P450 1A2 (CYP1A2), and 10% by cytochromes CYP2C9 and CYP2C19 [3], where it undergoes high first-pass hepatic metabolism. Agomelatine has at least four main metabolites, none of which have shown any toxic effects. Up to 80% of agomelatine is eliminated in urine as pharmacologically inactive metabolites, the main metabolites being hydroxylated and demethylated agomelatine [2,3].

#### Possible mechanism of agomelatine-induced liver injury

According to Gahr and colleagues [20], the mechanism underlying the agomelatine-induced liver injury appears to be idiosyncratic. This type of reactions occurre in most instances 5-90 days after the causative medication was last taken [21]. The mechanism of injury can be hepatocellular (predominant rise in alanine aminotransferase [ALT]), cholestatic (predominant rise in alkaline phosphatase [ALP]) or mixed [21].

## Review

### Adverse drug reactions: liver injury and agomelatine

Findings from the pre- and postmarketing clinical trials and non-interventional studies reporting agomelatine-induced liver injury are synthesized in Table 1.

**Table 1:** **Agomelatine-induced liver injury in pre- and postmarketing clinical trials and non-interventioal studies**

**Study [reference number]**	**No. Patients enrolled**	**Duration (weeks)**	**Indication**	**Liver enzymes >3X ULN**	**Serious hepatic reactions (i.e., hepatitis, liver enzymes >10X ULN and hepatic failure) and deaths due to hepatic reaction**
European clinical trials (2008) [8]	4068^1^	up to 52 weeks	All indications (Overall safety set)	Ago25: 1.04%^2^ Ago50: 1.39%^2^ Pbo: 0.72%	• >10X ULN: 1:1000-10 000^3^ 1 case hepatitis^4^
Zajecka et al. (2010) [22,23]	511	8	MDD	Ago25: 0% Ago50: 4.5% Pbo: 0%	0%
Stahl et al. (2010) [24,25]	503	8	MDD	Ago25: 0.6% Ago50: 3.0% Pbo: 0.6%	0%
CAGO178A2303 [26]	503	8	MDD	Ago25: 1.3% Ago50: 0.6% Par20: 0.6% Pbo: 0%	0%
Hale et al. (2010) [27]	515	8	MDD	Ago25/50: 1.6%^5^ Flx20/40: 0.4%	0%
Kasper et al. (2010) [28]	313	6	sleep in MDD	Ago25/50: 0.7%^5^ Ser50/100: 0%	0%
CAGO178A2301E1 (2009) [29]	329	52	MDD	Ago25/50: 2.0%	0%
CAGO178A2302E1 (2009) [30]	358	52	MDD	Ago25/50: 2.0%	0%
CAGO178A2303E1 (2010) [31]	334	52	MDD	Ago25: 1.2%^6^ Ago50: 2.0%^6^	0%
CAGO178A2304 [32]	633	52	MDD	Ago25/50: 4.6% (open-label phase) Pbo: 2.1% (double-blind continuation phase)^7^	0%
Calabrese et al. (2007) [33]	21	6, plus optional 46 (total 52)	bipolar I disorder	Ago25: N/A	0%
Olié & Kasper (2007) [34]	238	6	MDD	Ago25/50 and Pbo: N/A	0%
Goodwin et al. (2009) [35]	339	34	MDD	Ago25/50 and Pbo: N/A	0%
Demyttenaere et al. (2013) [36]	862^8^	up to 24	MDD	Ago25: 1.79% Ago50: 2.61% SSRIs: 0.34%	0%
Bruno et al. (2013) [37]	15	12	Fibromyalgia	Ago25: 0%	0%
Martinotti et al. (2012) [38]	60	8	MDD	Ago25/50 and Vlx75/150: N/A	0%
Stein et al. (2012) [39]	477	43	GAD	N/A	0%
Stein et al. (2014) [40]	412	12	GAD	N/A	0%
Heun et al. (2013) [41]	222	8	MDD	Ago25/50: 1.3%^5^ Pbo: 0%	0%
Laux and the VIVALDI Study Group (2012) [42]	3356	12	MDD	Ago25/50: 0.2%^8^	0%
Sparshatt et al. (2013) [43]	110	12	MDD	Ago25/50: -Baseline: 0.9%^9^ -Week 8: 0%	0%
Karaistos et al. (2013) [44]	40	16	Depression in type II diabetes	Ago25/50 and Ser50/100: N/A	0%
Quera-Slava et al. (2011) [45]	138	24	MDD	Ago25/50 and Esci10/20: N/A	0%
Montejo et al. (2010) [46]	92	8	healthy volunteers	Ago25/50, Par20, and Pbo: N/A	0%

Ago25 = 25 mg agomelatine; Ago50 = 50 mg agomelatine; Ago25/50 = entire agomelatine treatment group; Pbo = placebo; Par20 = 20 mg paroxetine, Flx20/40 = entire fluoxetine treatment group; Ser50/100 = entire sertraline treatment group; Vlx75/150 = entire venlafaxine treatment group; Esci10/20 = entire escitalopram treatment group; SSRIs: selective serotonin reuptake inhibitors; MDD = Major Depressive Disorder; GAD = Generalized Anxiety Disorder; Slp = sleep; N/A = non-vailable.

^1^: Number of patients receiving agomelatine; the number of patients receiving placebo is not clear.

^2^: Classified as a common event.

^3^: Classified as a rare event.

^4^: Case did not recover at follow-up (2.5 years after end of study).

^5^: Percentage calculated from safety set (i.e., enrolled patients who received at least one dose of the study treatment).

^6^: Percentage calculated from all treated patients enrolled in the extension phase.

^7^: Percentages calculated from information provided in Novartis Clinical Trial Results Database, 2010—study CAGO178A2.

304.

^8^: It is not mentioned by the authors if other ADs were taken concurrently.

^9^: Percentage calculated from information provided in the article.

#### Premarketing studies

##### European clinical trials

A pooled analysis of all completed Phase II/III European clinical trials of all indications (i.e., Overall Safety Set) was completed by the European Medicines Agency [8]. The analysis included a total of 4068 patients who received agomelatine, but it is not clear how many patients who received placebo were included. Significant elevations (>3X ULN) in alanine aminotransferase (ALT) and/or asparate aminotransferase (AST) liver enzymes in patients with normal values at baseline were reported at a rate of one in every 10-100 patients, rendering this finding a common occurrence. Specifically, significant elevations in liver enzymes occurred in 1.04% of patients taking 25 mg agomelatine, 1.39% taking 50 mg, and 0.72% taking placebo. All liver reactions observed were hepatocellular and occurred early during the six-month treatment period. Most reactions recovered within a few weeks, while others recovered during continued treatment or upon treatment discontinuation. Hepatic reactions occurring after three or six months of treatment were also observed, with no exact data given. Furthermore, rare cases of serious hepatic reactions (e.g., cytolytic hepatitis) have also been reported to have occurred at a rate of one in every 1000-10 000 persons [2,8]. Liver enzyme elevations of >10X ULN have also occurred, with no exact data available [8].

##### Short-term, multicenter, USA clinical trials

In an eight-week phase III clinical trial study by Zajecka and colleagues [22,23], no significant elevations in liver enzymes occurred in patients receiving either 25 mg agomelatine or placebo, but did occur in seven patients (4.5%) receiving 50 mg agomelatine. All liver reactions occurred within the sixth and eighth weeks of treatment. One patient discontinued treatment and their liver function tests normalized, and the other six patients continued treatment and their liver function tests normalized. The authors indicate that baseline hepatobiliary disorders (mainly in the form of cholecystitis, gallbladder disorder, and hepatic steatosis) existed in 3.1% of patients taking 50 mg agomelatine, in 0.6% taking 25 mg, and 0.6% receiving placebo. However, the authors do not indicate whether the existence of hepatobiliary disorders was associated with the significant increases in liver enzymes observed.

In a second eight-week clinical trial study by Stahl and colleagues [24,25], significant elevations in liver enzymes were noted in one patient (0.6%) taking 25 mg agomelatine, in five patients (3.0%) taking 50 mg agomelatine, and in one patient (0.6%) taking placebo. Again, most liver reactions occurred within the sixth and eighth weeks of treatment. Two agomelatine (50 mg) patients discontinued treatment and their liver function tests normalized, and four patients continued treatment during a one-year extension phase and their liver function tests normalized. One agomelatine patient (25 mg) was lost to follow-up.

In an unpublished eight-week phase III clinical trial [26], significant elevations in liver enzymes were found to occur in three patients (1.9%) taking agomelatine (two patients taking 25 mg agomelatine, and one patient taking 50 mg agomelatine), and in one patient (0.6%) taking 20 mg paroxetine. No patient receiving placebo developed clinically notable elevations in liver enzymes. One agomelatine (25 mg) patient discontinued treatment and their liver function tests normalized one day after discontinuation. It is mentioned that this patient had consumed alcohol the day before elevated liver enzymes were reported. The two other agomelatine patients continued treatment and their liver functions tests normalized. The patient receiving paroxetine continued treatment with agomelatine in an open-label extension phase and their liver function tests normalized.

##### Short-term multinational clinical trials

Hale et al. [27] compared agomelatine (25/50 mg) to fluoxetine (20/40 mg) during eight-weeks in outpatients diagnosed with MDD. Significant liver enzyme elevations were noted in four patients (1.6%) receiving agomelatine and in one patient (0.4%) receiving fluoxetine (percentages calculated using the safety set; i.e. all enrolled patients who received at least one dose of the study treatment). It is not indicated whether patients received 25 mg or 50 mg agomelatine. All patients continued treatment and their liver enzyme values normalized, in one case during the study treatment.

In a six-week study by Kasper et al. [28], where patients with MDD received either agomelatine (25/50 mg) or sertraline (50/100 mg), significant elevations in liver enzymes were noted in one patient (0.7%) receiving agomelatine (dosage not mentioned; percentage calculated using the safety set). The authors describe this patient as an alcoholic, and no additional information is provided.

##### Long-term efficacy and tolerability studies

In the unpublished 52-week, open-label extension phase study [29] to the published study by Zajecka et al. [22], six patients (2.0%) showed significant liver enzyme elevations. Specifically, three patients showed significant elevations in ALT values. All significant values normalized; two patients discontinued treatment and one patient recovered while on treatment. The other three patients showed significant elevations in gamma-glutamyl transferase (GGT) values. No additional information is provided concerning these patients. Lastly, one death occurred, with liver injury not being the cause of death [H. Maul, personal communication].

In a similar 52-week, open-label extension phase study to the published study by Stahl et al. [24,30], seven patients (2.0%) developed significant liver enzyme elevations. Of these patients, it is only mentioned that three recovered (two after treatment discontinuation and one while on treatment). No information regarding the other four patients is provided. Again, one death occurred, with liver injury not being the cause of death [H. Maul, personal communication].

Lastly, in another unpublished 52-week, open-label extension phase study to the study numbered CAGO178A2303 [26,31], one patient taking 25 mg agomelatine and five patients taking 50 mg agomelatine developed significantly elevated liver enzymes. Based on all treated patients in the extension phase, this would equal to 1.2% taking 25 mg agomelatine and to 2.0% taking 50 mg agomelatine. No additional information is provided. No deaths occurred during the extension phase.

##### Relapse prevention studies

Data from a 52-week, randomized, double-blind, placebo-controlled, relapse prevention study is also available [32]. Following an initial 16-24 weeks of open-label treatment with agomelatine (25/50 mg), 29 patients (4.6%; specific dosage not reported) developed liver enzyme elevations. During the double-blind treatment phase, three patients (2.1%) receiving placebo developed liver enzyme elevations). In neither treatment phase is it mentioned whether these elevations were significant. However, according to additional information received from Servier Labolatories, the reported elevations were not significant [H. Maul, personal communication].

##### Other studies

The following three studies were included, as blood samples were taken as part of the overall safety measures, and as the results from these blood samples are reported. However, it is not clear whether liver function tests were specifically performed.

Calabrese et al. [33] tested the AD efficacy of 25 mg agomelatine in patients with bipolar I disorder in an initial six-week open-label treatment phase followed by an optional extension phase up to 46 weeks (52 weeks total). The authors note that the results of all biochemical and blood parameters were not clinically relevant.

Olié and Kasper [34] compared agomelatine (25/50 mg) to placebo in an international, prospective, randomized, double-blind, parallel-group study in MDD patients. Results of the biochemical variables examined are not specifically reported by the authors, but they do mention that no relevant differences between treatment groups were observed in any of the biochemical variables examined during treatment. The authors further mention that the most common reason for study discontinuation was lack of treatment efficacy. Taken together, this information likely suggests that significantly elevated liver enzymes were not observed during the study period.

In a multinational, randomized, double-blind, placebo-controlled, relapse prevention study by Goodwin et al. [35] MDD patients who responded to an initial eight or ten-week agomelatine treatment period received either agomelatine (25/50 mg) or placebo for an additional 24 weeks. No differences between treatment groups were observed from baseline to the end of the treatment in any of the haematological and biochemical variables examined.

#### Postmarketing studies

In an update from the Medicines and Healthcare products Regulatory Agency in 2012 [9], it is mentioned that six reported cases worldwide of hepatic failure [rate of one in every 1000-10 000 persons; 10] have occurred since Servier was granted marketing authorization of agomelatine in 2009, resulting in death or liver transplantation in people with hepatic risk factors. Serious hepatic reactions including hepatitis (cytolytic) and jaundice, and elevations of liver enzymes >10X ULN have also been reported, with no exact frequency data given [10]. It is mentioned however that most reactions occurred during the first months of treatment, were hepatocellular, and that liver enzymes usually normalized upon treatment cessation. Only one case of hepatitis did not recover at follow-up after discontinuation of agomelatine (2.5 years after the end of the study) is reported.

Data from clinical trials examining the long-term safety of agomelatine compared to other ADs have also recently become available via the publication of a meta-analysis by Demyttenaere et al. [36]. In their analysis of four clinical trials with an extension phase of up to 24 weeks, the authors note that significant elevations in liver serum transaminase values occurred in eight patients (1.75%) receiving 25 mg agomelatine, in four patients (2.61%) receiving 50 mg agomelatine and in two patients (0.34%) receiving a comparative AD (either fluoxetine, sertraline or escitalopram) during the extension phase. These results are similar to those reported in short-term clinical trials and in the pooled analysis of all completed Phase II/III European clinical trials by the European Medicines Agency (European Medicines Agency [8].

##### European clinical trials

Bruno et al. [37] examined the efficacy and tolerability of agomelatine (25 mg) in the treatment of fibromyalgia in a twelve-week, open-label study. Liver function tests were measured at inclusion and upon completion of the study. Twelve females completed the study, and none showed significant changes in any of the laboratory parameters examined.

In an eight-week, open-label pilot study by Martinotti et al. [38] comparing agomelatine (25/50 mg) and venlafaxine (75/150 mg) in the treatment of anhedonia in MDD, liver enzyme values were examined at baseline and at the end of the study. The authors report that no clinically relevant differences were seen between treatment groups in the mean change from baseline in liver enzyme values.

##### Multinational trials

In a six-month, randomized, double-blind, placebo-controlled study in patients with Generalized Anxiety Disorder (GAD) by Stein et al. [39], it is mentioned that potentially clinically significant abnormal liver enzyme values were noted in 17 patients (3.6%). Specifically, ten patients received 25 mg agomelatine, 3 patients received 50 mg agomelatine, and 5 patients received placebo; one patient receiving 25 mg agomelatine continued to show elevated liver enzyme values when switched to placebo in the maintenance period of the study. Here, it is not possible to calculate percentages per group (i.e., agomelatine 25, agomelatine 50, and placebo), as information regarding total group numbers is lacking. The authors additionally mention that all abnormal values normalized during the treatment period, except for total bilirubin values in three patients receiving placebo. No patient receiving agomelatine terminated treatment due to adverse events, whereas two patients receiving placebo did during the maintenance period. It is not clear whether this was specifically due to significant liver enzyme elevations.

In a similar study by Stein et al. [40] in outpatients diagnosed with GAD who received agomelatine (25/50 mg), escitalopram (10/20 mg) or placebo, two patients (1.4%) receiving agomelatine and two patients (1.4%) receiving escitalopram showed significant elevations in liver enzymes (percentages calculated from safety set). The dosage these patients received is not indicated. No patient in the placebo group showed significant liver enzyme elevations. No patient discontinued the study due to significantly elevated liver enzymes, and the authors mention that all liver function tests normalized upon treatment discontinuation.

In an eight-week study in elderly MDD outpatients (aged ≥ 65 years) comparing agomelatine (25/50 mg) to placebo by Heun et al. [41], two patients (1.3%; percentage calculated from safety set) receiving agomelatine (one aged ≥ 75 years; specific dosage not reported) with normal liver enzyme values at baseline showed significant elevations in liver enzymes during the study treatment. The authors mention that these reactions were likely due to agomelatine and that all abnormal values normalized upon treatment discontinuation.

##### Non-interventional studies in routine practice

In a 12-week study of patients by Laux and the VIVALDI Study Group 2 [42], significant elevations in liver enzymes specifically reported as an adverse drug reaction were noted in eight patients (0.2%). For five patients, it was possible for the authors to assess the evolution of abnormal liver enzyme values as both baseline and at least one follow-up value were available. In short, two patients (0.1%) were found to have normal ALT values at baseline that significantly increased during the study period, and two patients (percentage not provided) had elevated, but not significantly elevated ALT values (i.e., >ULN and ≤ 3X ULN) at baseline that reached significant levels during the study period. Two of these four patients had normal AST values at baseline that also significantly increased during the study. Finally, one patient (percentage not provided) showed significant AST elevations, but no significant ALT elevations during the observation period. For all information regarding significant liver enzymes, it is not clear whether patients received 25 mg or 50 mg agomelatine, and whether patients received other concurrent medication(s). It is also not specified when these liver reactions occurred during treatment, whether any of these patients discontinued treatment, and whether liver function tests normalized upon treatment cessation.

Sparshatt and colleagues [43] also conducted a 12-week non-interventional study with difficult-to-treat or refractory MDD patients. At baseline (99.1% taking 25 mg agomelatine), liver enzyme values for three patients (3.6%) were outside the normal reference range, but all were within 3XULN range, and one patient’s (0.9%; percentages calculated from information provided in the article) liver enzyme values were significantly elevated. This patient discontinued treatment, with the prescriber stating “liver disorder” as the reason for treatment discontinuation. However, as the abnormal liver enzyme values were noted at baseline, it is unlikely that agomelatine treatment resulted in the observed finding. At week eight, results from 13 patients (14.9%) were outside the normal reference range, but none were significantly elevated. Of these patients, ten continued treatment (of whom two had abnormal values at baseline) and three discontinued treatment due to a lack of efficacy. It should be noted that 23 patients (20.1%) received at least one additional AD in addition to agomelatine at baseline. Thus, establishing causality between drug treatment and hepatotoxicity is rendered more complex. Lastly, it is mentioned that liver function tests were performed at week 12, but the results from these tests are not provided.

Karaiskos et al. [44] compared agomelatine (25/50 mg) and sertraline (50/200 mg) for the treatment of depression in type II diabetes. Liver function tests were performed at baseline and after six weeks of treatment. The authors mention that both treatments were well tolerated, and that no patient dropped-out of the study. No further details are provided.

##### Other studies

As in section 1.6, the following two studies were included as blood samples were taken as part of the overall safety measures, and as the results from these blood samples are reported. However, it is likewise not clear if liver function tests were specifically performed.

In a 24-week multinational, multicentre sleep study by Quera-Slava et al. [45], agomelatine (25/50 mg) and escitalopram (10/20 mg) were compared in terms of AD efficacy, as well as regarding their effects on nighttime sleep and daytime condition. Measures of haematology and biochemistry were tested at baseline, and at weeks six and twelve. No clinically relevant findings were observed.

In an eight-week study comparing the effects of agomelatine (25/50 mg), paroxetine (20 mg) and placebo on measures of sexual acceptability in healthy male subjects by Montejo et al. [46], the authors report that no clinically relevant changes in mean values of biochemical and blood parameters were observed.

##### Case report

We found one published case report of agomelatine-induced liver injury [47]. After 3 weeks of agomelatine treatment (dose increased from 25 mg to 50 mg in one week; no concurrent medications listed), a female patient aged 44 years old showed significant elevations in liver enzyme. The author indicates that the patient did not have a history of liver disease and that their liver function tests at baseline were within the normal range. The type of liver damage observed was hepatocellular, and the patient’s liver enzyme values returned to normal upon agomelatine discontinuation.

##### Pharmacovigilance database reviews

Gahr et al. [20] analysed reports of agomelatine-related liver injury submitted to a German pharmacovigilance database. 58 cases were submitted, most of which (79%) being asymptomatic increases in liver enzymes. Six cases (10%) of agomelatine-related hepatitis were also found. In their analysis, the authors mention that the female gender, increased age (mean 48.5 years; range 39-58), and polypharmacy may be risk factors for agomelatine-related hepatotoxicity.

Likewise, Montastruc and colleagues [48] analysed the overall prevalence of agomelatine-related liver injury, compared to that of 17 other ADs using data from Spanish, French, Italian, and Portuguese pharmacovigilance databases. In total, 3300 cases of AD-related liver injury were reported and collected from the databases surveyed, accounting for 10.3% of all cases collected for these drugs. The authors further report that a total of 63 cases of agomelatine-related liver injury were found since its market introduction until the end of the study period, accounting for 14.6% of AD-related liver injury compared to the other ADs included in their review. Agomelatine was further found to be statistically associated with liver injury in Spain, France and Italy. As mentioned by the authors, the different prevalence rates found for all drugs combined in comparison to agomelatine might be explained by different guidelines regarding the reporting of adverse drug events for different drugs to pharmacovigilance databases. Another explanation provided by the authors concerns differences in the prescription of ADs in terms of patient population and duration.

### Other considerations

#### Dose-effect relationship

Findings supporting the claim that increased drug dosage increases the risk of DILI have been observed in the premarketing trials of agomelatine. Based on this review, it is harder to establish such a relationship in the postmarketing setting as results from most studies are reported for the entire agomelatine group (i.e., 25/50 mg combined). However, one could hypothesize the existence of such a relationship (see [49]).

#### Time of treatment

It is not clear whether and if so, to what extent, a risk of liver injury under agomelatine treatment changes in time. Various studies presented here report the significant increase of liver enzymes occurs most often in the first weeks of treatment. As already mentioned, the idiosyncratic reactions, which are currently assumed to underly the agomelatine-induced liver injury, most often occur in 5-90 days after the last administration of the causative medication [21]. The various currently available reports on the prevalence of side effects including agomelatine-related liver injury cover a pre-defined treatment period of 6 to 52 weeks (compare Table 1). However, our search did not provide any trials looking at the risk of liver injury in patients treated with agomelatine over a time period longer than one year. Taking into consideration the fact that antidepressant medication is usually given as a chronic or long-time treatment, conducting long-time observations (over 2 and more years preferably) would provide key information with regard to the long-time drug safety and undoubtedly be welcomed by clinicians.

#### Special populations

##### Impaired liver function

No data from patients with hepatic insufficiency (i.e., cirrhosis or other active liver diseases) are available in Phase II/III studies as this clinical condition was an exclusion criterion [8]. However, an open-study (PKH-014) was performed to evaluate the influence of hepatic insufficiency in patients with mild (Child-Pugh grade A) or moderate (Child-Pugh grade B) liver failure due to alcohol cirrhosis after taking a single dose of 25 mg agomelatine [8]. On average, the increase in agomelatine exposure was 70-fold higher in patients with mild liver insufficiency compared to healthy controls, and 140-fold higher in patients with moderate liver insufficiency compared to healthy controls. Thus, liver insufficiency leads to a significant increase in agomelatine exposure, and therefore agomelatine is contraindicated in patients with a hepatic insufficiency.

##### Elderly and youth

As agomelatine has not been found to be effective in elderly patients (≥75 years) diagnosed with MDD, it should not be prescribed to patients in this age group [8]. However, findings from Heun et al. [41] highlight the need to further evaluate the efficacy of agomelatine in MDD patients aged ≥65 years, as agomelatine was found to be effective in this age group—although no real value was found in a subgroup of patients aged ≥75. Also, as mentioned previously, Heun et al. [41] did note significant liver enzyme elevations in two patients during their 8-week study (one patient aged ≥75 years). More data would thus be welcomed to additionally gain a better understanding of agomelatine-related liver injury in this age group.

In addition, agomelatine is not recommended for children and adolescents under the age of 18 suffering of MDD as no clinical trials were conducted in this population [8].

#### Drug-drug interactions

In a study investigating the combined administration of fluvoxamine (a potent CYP1A2 inhibitor) and agomelatine, a 60-fold increase in the drug concentration over time (or AUC, for area under the curve) was shown [8]. Indeed, fluvoxamine inhibits the metabolism of agomelatine, resulting in an increased exposure to agomelatine. Therefore, the concomitant administration of agomelatine with potent CYP1A2 inhibitors (e.g., fluvoxamine or ciprofloxacin) is contraindicated. Caution is furthermore recommended when prescribing agomelatine with moderate CYP1A2 inhibitors (e.g., propanolol, grepafloxacin, and enoxacin). Findings from Gahr and colleagues [20] likewise highlight polypharmacy as a potential risk factor for agomelatine-induced hepatotoxicity.

## Discussion

To our knowledge, this is the first review that solely provides an in-depth evaluation of agomelatine-induced liver injury compared to placebo and the four active comparator AD treatments used in the clinical trials for agomelatine by reviewing findings from both the pre- and postmarketing settings, and by including published and unpublished studies. Findings of agomelatine-induced liver injury from non-interventional studies, pharmacovigilance database reviews and one case report are also reported.

When combining findings from pre- and postmarketing clinical trials, we find that significant elevations in liver enzymes (>3X ULN) have been found to occur at a rate of up to 4.6% (25/50 mg) following the administration of agomelatine (see study CAGO178A2304; [33]). This is in-line with the information found in the Summary of Product Characteristics for agomelatine, where significant liver enzyme elevations are noted to have occurred at a rate of one in every 10-100 patients during the clinical trials of agomelatine [2]. Findings from the European premarketing clinical trials furthermore indicate that the risk of agomelatine-induced liver injury increases with increasing dosage, where 1.04% of patients treated with 25 mg/day reported significantly elevated liver enzyme values compared to 2.5% of patients treated with 50 mg/day and 0.6% receiving placebo [8,50].

However, we have also seen that receiving a placebo as well as any of the four active comparator ADs selected in the clinical trials for agomelatine poses a risk of inducing liver injury at similar rates—but on average less—compared to agomelatine. Indeed, rates of liver injury were found to have occured at a rate as high as 2.1% for placebo, 1.4% for escitalopram, 0.6% for paroxetine, 0.4% for fluoxetine, and 0% for sertraline. Although not compared in this review, it is worth noting that Voican et al. [7] recently highlighted the increased risk of liver injury following the administration of several commonly prescribed AD treatments, with rates higher or very similar to those of agomelatine. To name a few, the authors list the ADs iproniazid, nefazodone, phenelzine, imipramine, and amitriptyline (for a complete list, see [7]). Likewise, the available AD duloxetine has recently come under scrutiny after 13 cases of death due to hepatic failure being reported [51]. The same review by Voican et al. [7] showed citalopram, escitalopram, paroxetine, and fluvoxamine seem to have the least potential for hepatotoxicity. At any rate, it is important to note that elevated, yet not clinically significant elevations in liver serum transaminase titers (i.e., <3X ULN) have been found to occur at a rate of approximately 1% to 5% within the general population [52]. Furthermore, liver enzyme values of <3X ULN have been noted to be observable in approximately 20% of individuals receiving placebo during phase 1 clinical trials [53]. Therefore, results showing clinically significant liver enzyme elevations from patients receiving AD-treatment should be interpreted with caution, as a baseline variability in liver enzyme values is observable in the general population.

Interestingly, we found a lower incidence rate of significantly elevated liver enzymes following the administration of agomelatine in non-interventional studies. Specifically, Laux and the VIVALDI Study Group [42] report a rate of 0.2% and Sparshatt et al. [43] report a baseline rate of 0.9%. However, as previously mentioned, it is unlikely that the administration of agomelatine caused the significantly elevated liver enzyme values observed in the study by Sparshatt et al. [43], as the abnormal values were reported at baseline. One hypothesis to explain the observed difference in significantly elevated liver enzyme values between clinical trials and non-interventional studies might be due to fundamental differences between these two types of studies. For example, clinical studies more intensively and systematically monitor adverse drug reactions as part of standard practice, whereas non-interventional studies generally rely more on spontaneous reports of adverse drug reactions between liver function tests, and may therefore miss cases of potential liver injury, as abnormal values tend to normalize within a few weeks [8]. At any rate, one could argue that findings from non-interventional studies provide data that is more representative of the general population than findings from clinical trials, as the inclusion criteria that participants have to meet in non-interventional studies tend to be less stringent than those of clinical trials.

Furthermore, findings from Gahr et al. [20] and Montastruc et al. [48] of pharmacovigilance databases help to contextualize the risk of agomelatine-induced liver injury in terms of reported frequency. At the time of their query, Gahr et al. [20] found that 10% of all submitted reports of agomelatine-related liver injury to the selected pharmacovigilance database were of significantly elevated liver enzyme values, whereas Montastruc et al. [48] found agomelatine to account for 10.3% of all cases of liver injury submitted to the selected pharmacovigilance databases they reviewed.

These are considerable amounts, but as mentioned by Gahr et al. [20], such findings cannot be interpreted as an estimation of the overall incidence rate of agomelatine-induced liver injury, as physicians are not required by law to report possible cases of adverse drug events to the responsible pharmacovigilance database they surveyed. It is not clear whether this is also the case in the study by Montastruc and colleagues [48]. However, Gahr et al. [20] interestingly mention that there is a trend of underreporting adverse drug events to pharmacovigilance institutions (see [54]). In any case, such data are helpful in identifying potential risk factors associated with agomelatine and liver injury, and here Gahr et al. [20] highlight the female gender, increased aged, and polypharmacy. Interestingly, two of these potential risk factors (i.e., female gender and increased age) figure in the one published case report of agomelatine-induced hepatotoxicity found during the writing of this article [47].

### Limitations and strengths

A main limitation of this review is related to publication bias. It is clear that case reports and studies of pharmacovigilance database reviews are inherently biased vis-à-vis the publication of more severe cases, and as mentioned previously, cannot be used as an estimate of incidence rates. Moreover, publication bias is also inherently present toward the publication of clinical trials where agomelatine has been found to be more effective and safer than placebo and/or other ADs. The inclusion of unpublished clinical trials therefore attempted to reduce this bias. A second limitation regards the inclusion of studies in which blood samples rather than specific tests of liver function being stated as part of the study’s safety procedure. Although only liver functions tests can properly assess a potential drug-induced liver injury [7], healthcare professionals prescribing agomelatine must routinely monitor patients’ liver function, and thus it seems unlikely that any case of significantly elevated liver enzyme values would not be reported. Furthermore, this review offers a qualitative summary of agomelatine-induced liver injury. A quantitative understanding of this relationship would benefit the current literature. However, because of the heterogeneity between studies, namely in patient inclusion criteria and study design, combining study results would prove to be a challenge and would likely lead to the exclusion of several studies included in this review. Likewise, a quantitative comparative assessment of agomelatine-induced liver injury compared to other commonly prescribed ADs (i.e., not limited to the four active comparator ADs used in the clinical trials for agomelatine) was beyond the scope of this review, and warrants further investigation.

The main strength of this review lies in the extensive inclusion and analysis of both published and unpublished clinical trials, non-interventional studies, pharmacovigilance database revies and case report for which agomelatine has been tested in diseases and conditions not limited to MDD. This allowed to have a broad overview of agomelatine-induced liver injury.

### Risk-benefit assessment

Given the considerable amount of AD treatments currently available, and the substantial heterogeneity in patient response to ADs, there is still a fundamental need to develop more efficient and especially better tolerated ADs. As mentioned in the assessment report for agomelatine by the Committee for Medicinal Products for Human Use [8], an effective AD with a more favourable safety profile compared to existing ADs—whether or not it is found to be more effective than these ADs—would still be considered a valuable addition to the current AD treatment armamentarium. This is especially relevant when one considers that poor compliance due to adverse drug reactions still remains one of the most common reasons for treatment discontinuation [22]. In this regard, agomelatine can be judged as a beneficial AD treatment. Specifically, its unique non-monoaminergic mechanism of action endows it with a different safety profile than other existing AD classes. Indeed, compared to other existing ADs, studies have shown that agomelatine poses a low risk of sexual dysfunction, low incidence of gastrointestinal reactions, lack of significant weight gain or serotonin syndrome, lack of discontinuation symptoms, and overall incidence rates of adverse events similar to those from placebo [8]. These characteristics are likely to be welcomed by patients, their families, and healthcare professionals.

In terms of liver injury, we have seen that ADs in general, including agomelatine, have the potential to cause liver damage. In this regard, the manufacturer of agomelatine proposes a detailed liver monitoring program, and upholds that the benefits of taking agomelatine outweigh its potential risk of hepatotoxicity if healthcare professional adhere to it. Interestingly, it has recently been argued that introducing amendments to the current threshold values for abnormal ALT and ALP tiers could provide more specific markers of drug-induced liver injury. For example, Verma and Kaplowitz [55] mention that the current threshold values (i.e., >3X ULN for ALT tiers and >2X ULN for ALP tiers) are sensitive, but not specific markers of liver injury. Thus, at least when considering ALT values, a new threshold value of 5XULN has been proposed to limit the withdrawal of drugs incorrectly identified as being hepatotoxic [56].

Lastly, it is worth mentioning that the metabolic processes of the liver, including drug metabolism, are affected by circadian rhythmicity [57]. As agomelatine is prescribed to be taken in the evening (ref), whereas many other ADs are to be taken during the day, considering the time of day at which ADs are taken and the resulting effects this has on drug metabolism and potential liver injury is a factor that warrants closer examination when comparing agomelatine to other ADs.

## Conclusions

Agomelatine prescription at therapeutic doses does pose a risk of inducing liver injury, which is usually reversible. However, rare cases of severe and life-threatening hepatotoxicity have also occurred. Therefore, it is essential that clinicians continue to monitor liver function frequently, as prescribed by the manufacturer of agomelatine. Early detection, followed by best practice treatment plan reactions (e.g., treatment discontinuation), remain the most efficient responses toward possible manifestations of liver damage. Informing patients that agomelatine can be associated with liver injury, that certain factors may increase one’s risk (e.g., concurrent alcohol use), and that various biological and clinical manifestations (e.g., fatigue, nausea, vomiting, dark urine, etc.) may indicate liver injury is of crucial importance.

### Consent

This review provides an analysis of the data already published and as such does not concern the publication of any other medical information or documentation from the patients. As a result no further informed consent was obtained.

## References

[1] Yous S, Andrieux J, Howell HE, Morgan PJ, Renard P, Pfeiffer B (1992). Novel naphthalenic ligands with high affinity for the melatonin receptor. J Med Chem.

[2] Servier Laboratories Limited: Summary of product characteristics: Valdoxan 25 mg film-coated tablets [http://www.medicines.org.uk/emc/medicine/21830/SPC/valdoxan/]

[3] Therapeutic Goods Administration, Department of Health and Ageing: Australian public assessment report for agomelatine [http://www.tga.gov.au/pdf/auspar/auspar-valdoxan.pdf]

[4] Taylor D, Sparshatt A, Varma S, Olofinjana O (2014). Antidepressant efficacy of agomelatine: meta-analysis of published and unpublished studies. BMJ.

[5] Guaiana G, Gupta S, Chiodo D, Davies SJC, Haederle K, Koesters M (2013). Agomelatine versus other antidepressive agents for major depression. Cochrane Database Syst Rev.

[6] Navarro V, Senior JR (2006). Drug-related hepatotoxicity. N Engl J Med.

[7] Voican CS, Corruble E, Naveau S, Perlemuter G (2014). Antidepressant-induced liver injury: a review for clinicians. Am J Psychiatry.

[8] European Medicines Agency: Evaluation of medicines for human use—CHMP assessment report for Valdoxan [http://www.ema.europa.eu/docs/en_GB/document_library/EPAR_-Public_assessment_report/human/000915/WC500046226.pdf]

[9] Drug safety update, Medicines and Healthcare products Regulatory Agency: Agomelatine (Valdoxan/Thymanax): risk of dose-related hepatotoxicity and liver failure—update warnings and monitoring guidance [http://www.mhra.gov.uk/home/groups/dsu/documents/publication/con199577.pdf]

[10] Servier Laboratories Limited: Agomelatine (Valdoxan): monitor liver function and do not use in people with high serum transaminase levels (>3 x ULN) or ≥ 75 years [http://www.servier.co.uk/pdfs/direct-healthcare-professional-communication.pdf]

[11] Temple RJ, Himmel MH (2002). Safety of newly approved drugs: implications for prescribing. JAMA.

[12] European Medicines Agency, Committee for Medicinal Products for Human Use: Reflection paper on non-clinical evaluation of drug-induced liver injury [http://www.ema.europa.eu/docs/en_GB/document_library/Scientific_guideline/2010/07/WC500094591.pdf

[13] Novartis clinical trial results database: CAGO178: Major Depressive Disorder (MDD) [http://wwctrdW/ctrdWebApp/clinicaltrialrepository/public/product.jsp?productID=268&diseaseAreaID=3]

[14] Antidepressiva Forum Deutschland Wissen: Agomelatin/Studien [http://www.adfd.org/wissen/Agomelatin/Studien]

[15] Li JZ, Bunney BG, Meng F, Hagenauer MH, Walsh DM, Vawter MP (2013). Circadian patterns of gene expression in the human brain and disruption in major depressive disorder. Proc Natl Acad Sci U S A.

[16] Audinot V, Mailliet F, Lahaye-Brasseur C, Bonnaud A, Le Gall A, Amossé C (2003). New selective ligands of human cloned melatonin MT1 and MT2 receptors. Naunyn Schmiedeberg’s Arch Pharmacol.

[17] Millan MJ, Gobert A, Lejeune F, Dekeyne A, Newman-Tancredi A, Pasteau V (2003). The novel melatonin agonist Agomelatine (S20098) is anantagonist at 5-hydroxytryptamine2C receptors, blockade of which enhances the activity of frontocortical dopaminergic and adrenergic pathways. J Pharmacol Exp Ther.

[18] Quera-Slava MA, Vanier B, Laredo J, Hartley S, Chapotot F, Moulin C (2007). Major depressive disorder, sleep EEG and agomelatine: an open-label study. Int J Neuropsychopharmacol.

[19] De Berardis D, Di Iorio G, Acciavatti T, Conti C, Serroni N, Olivieri L (2011). The emerging role of melatonin agonists in the treatment of major depression: Focus on agomelatine. CNS Neurol Disord Drug Targets.

[20] Gahr M, Freudenmann RW, Connenmann BJ, Hiemke C, Schönfeldt-Lecuona C (2013). Agomelatine and hepatotoxicity: implications of cumulated data derived from spontaneous reports of adverse drug reactions. Pharmacopsychiatry.

[21] Hussaini SH, Farrington EA (2007). Idiosyncratic drug-induced liver injury: an overview. Expert Opin Drug Saf.

[22] Zajecka J, Schatzberg A, Stahl S, Shah A, Caputo A, Post A (2010). Efficacy and safety of agomelatine in the treatment of major depressive disorder: a multicenter, randomized, double-blind, placebo-controlled trial. J Clin Psychopharmacol.

[23] Novartis clinical trial results database, Study number CAGO178A2301: An 8-week, randomized, double-blind, fixed dosage, placebo-controlled, parallel-group, multi-center study of the efficacy, safety and tolerability of agomelatine 25 mg and 50 mg in the treatment of Major Depressive Disorder (MDD). [http://wwctrdW/ctrdWebApp/clinicaltrialrepository/public/product.jsp?productID=268&diseaseAreaID=3]

[24] Stahl S, Fava M, Trivedi MH, Caputo A, Shah A, Post A (2010). Agomelatine in the treatment of Major Depressive Disorder: an 8-week, multicenter, randomized, placebo-controlled trial. J Clin Psychiatry.

[25] Novartis clinical trial results database, Study number CAGO178A2302: An 8-week, randomized, double-blind, fixed dosage, placebo-controlled, parallel-group, multi-centerstudy of the efficacy, safety and tolerability of agomelatine 25 mg and 50 mg in the treatment of Major Depressive Disorder (MDD) [http://wwctrdW/ctrdWebApp/clinicaltrialrepository/public/product.jsp?productID=268&diseaseAreaID=3]

[26] Novartis clinical trial results database, Study number CAGO178A2303: An 8-weel, multicenter, randomized, double-blind, placebo- and paroxetine-controlled study of the efficacy, safety and tolerability of agomelatine 25 or 50 mg given once daily in the treatment of Major Depressive Disorder (MDD) [http://www.novctrd.com/ctrdWebApp/clinicaltrialrepository/public/product.jsp?productID=268&diseaseAreaID=3]

[27] Hale A, Corral RM, Mencacci C, Ruiz JS, Severo CA, Gentil V (2010). Superior efficacy results of agomelatine versus fluoxetine in severe MDD patients: a randomized, double-blind study. Int Clin Psychopharmacol.

[28] Kasper S, Hajak G, Wulff K, Hoogendijk WJC, Montejo AL, Smeraldi E (2010). Efficacy of the novel antidepressant agomelatine on the circadian rest-activity cycle and depressive and anxiety symptoms in patients with Major Depressive Disorder: a randomized, double-blind comparison with sertraline. J Clin Psychiatry.

[29] Novartis clinical trial results database, Study number CAGO178A2301E1: A 52-week, open-label extension to an 8-week, randomized, double-blind, fixed dosage, placebo-controlled, parallel-group, multi-center study of the efficacy, safety and tolerability of agomelatine 25 mg and 50 mg in the treatment of Major Depressive Disorder [http://www.novctrd.com/ctrdWebApp/clinicaltrialrepository/public/product.jsp?productID=268&diseaseAreaID=3]

[30] Novartis clinical trial results database, Study number CAGO178A2302E1: A 52-week, open-label extension to an 8-week, randomized, double-blind, fixed dosage, placebo-controlled, parallel-group, multi-center study of the efficacy, safety and tolerability of agomelatine 25 mg and 50 mg in the treatment of Major Depressive Disorder [http://www.novctrd.com/ctrdWebApp/clinicaltrialrepository/public/product.jsp?productID=268&diseaseAreaID=3]

[31] Novartis clinical trial results database, Study number CAGO178A2303E1: A 52-week, open-label extension to Study AGO178A2303, an 8-week, multicenter, randomized, double-blind, placebo- and paroxetine-controlled study of the efficacy, safety and tolerability of agomelatine 25 or 50 mg given once daily in the treatment of Major Depressive Disorder [http://www.novctrd.com/ctrdWebApp/clinicaltrialrepository/public/product.jsp?productID=268&diseaseAreaID=3]

[32] Novartis clinical trial results database, Study number CAGO178A2304: A 52-week, randomized, double-blind, placebo-controlled, multicenter, parallel-group study of the long-term efficacy, tolerability and safety of agomelatine 25 and 50 mg in the prevention of relapse of Major Depressive Disorder (MDD) following open-label treatment of 16-24 weeks [http://www.novctrd.com/ctrdWebApp/clinicaltrialrepository/public/product.jsp?productID=268&diseaseAreaID=3]

[33] Calabrese JR, Guelfi JD, Perdrizet-Chevallier C, Agomelatine Bipolar Study Group (2007). Agomelatine adjunctive therapy for acute bipolar depression: preliminary open data. Bipolar Disord.

[34] Olié JP, Kasper S (2007). Efficacy of agomelatine, a MT1/MT2 receptor agonist with 5-HT_2C_ antagonistic properties, in major depressive disorder. Int J Neuropsychopharmacol.

[35] Goodwin GM, Emsley R, Rembry S, Rouillon F (2009). Agomelatine prevents relapse in patients with Major Depressive Disorder without evidence of a discontination syndrome: a 24-week randomized, double-blind, placebo-controlled trial. J Clin Psychiatry.

[36] Demyttenaere K, Corruble E, Hale A, Quera-Slava MA, Picarel-Blanchot F, Kasper S (2012). A pooled analysis of six month comparative efficacy and tolerability in four randomized clinical trials: agomelatine versus escitalopram, fluoxetine, and sertraline. CNS Spectr.

[37] Bruno A, Micò U, Lorusso S, Cogliandro N, Pandolfo G, Caminiti M (2013). Agomelatine in the treatment of fibromyalgia: a 12-week, open-label, uncontrolled preliminary study. J Clin Psychopharmacol.

[38] Martinotti G, Sepede G, Gambi F, Di Iorio G, De Berardis D, Di Nicola M (2012). Agomelatine versus venlafaxine XR in the treatment of anhedonia in major depressive disorder: a pilot study. J Clin Psychopharmacol.

[39] Stein D, Ahokas A, Albarran C, Olivier V, Allgulander C (2012). Agomelatine prevents relapse in Generalized Anxiety Disorder: a 6-month randomized, double-blind, placebo-controlled discontinuation study. J Clin Psychiatry.

[40] Stein DJ, Ahokas A, Marquez M, Höschl C, Oh KS, Jarema M (2014). Agomelatine in Generalized Anxiety Disorder: an active comparator and placebo-controlled study. J Clin Psychiatry.

[41] Heun R, Ahokas A, Boyer P, Giménez-Montesinos N, Pontes-Soares F, Olivier V (2013). The efficacy of agomelatine in elderly patients with recurrent Major Depressive Disorder: a placebo-controlled study. J Clin Psychiatry.

[42] Laux G, VIVALDI Study Group (2012). The antidepressant agomelatine in daily practice: results of the non-interventional study VIVALDI. Pharmacopsychiatry.

[43] Sparshatt A, McAllister WRH, Baldwin DS, Haddad PM, Bazire S, Weston E (2013). A naturalistic evaluation and audit database of agomelatine: clinical outcome at 12 weeks. Acta Psychiatr Scand.

[44] Karaiskos D, Tzavellas E, Illias I, Liappas I, Paparrigopoulos T (2013). Agomelatine and sertraline for the treatment of depression in type 2 diabetes mellitus. Int J Clin Pract.

[45] Quera-Salva MA, Hajak G, Philip P, Montplaisir J, Keufer-Le Gall S, Laredo J (2011). Comparison of agomelatine and escitalopram on nighttime sleep and daytime condition and efficacy in major depressive disorder patients. Int Clin Psychopharmacol.

[46] Montejo AL, Prieto N, Terleira A, Matias J, Alonso S, Paniagua G (2010). Better sexual acceptability of agomelatine (25 and 50 mg) compared with paroxetine (20 mg) in healthy male volunteers. An 8-week, placebo-controlled study using the PRSEXDQSALSEX scale. J Psychopharmacol.

[47] Štuhec M (2013). Agomelatine-induced hepatotoxicity. Wien Klin Wochenschr.

[48] Montastruc F, Scotto S, Vaz IR, Guerra LN, Escudero A, Sainz M (2014). Hepatotoxicity related to agomelatine and other new antidepressants: a case/noncase approach with information from the Portuguese, French, Spanish, and Italian pharmacovigilance systems. J Clin Psychopharmacol.

[49] Lammert C, Einarsson S, Saha C, Niklasson A, Bjornsson E, Chalasani N (2008). Relationship between daily dose of oral medications and idiosyncratic drug-induced liver injury: search for signals. Hepatology.

[50] Howland RH (2011). A benefit-risk assessment of agomelatine in the treatment of major depression. Drug Saf.

[51] McIntyre RS, Panjwani ZD, Nguyen HT, Woldeyohannes HO, Alsuwaidan M, Soczynska JK (2008). The hepatic safety profile of duloxetine: a review. Expert Opin Drug Metab Toxicol.

[52] Neuschwander-Tetri BA, Caldwell SH (2003). Nonalcoholic steatohepatitis: summary of an AASLD Single Topic Conference. Hepatology.

[53] Rosenzweig P, Miget N, Brohier S (1999). Transaminase elevation on placebo during Phase I trials: prevalence and significance. Br J Clin Pharmacol.

[54] Jacob D, Marrón B, Ehrlich J, Rutherford PA (2013). Pharmacovigilance as a tool for safety and monitoring: a review of general issues and the specific challenges with end-stage renal failure. Drug Healthc Patient Saf.

[55] Verma S, Kaplowitz N (2009). Diagnosis, management, and prevention of drung-induced liver injury. Gut.

[56] Aithal GP, Watkins PB, Andrade RJ, Larrey D, Molokhia M, Takikawa H (2011). Case definition and phenotype standardization in drug-induced liver injury. Clin Pharmacol Ther.

[57] Gachon F, Firsov D (2011). The role of circadian timing system on drug metabolism and detoxification. Expert Opin Drug Metab Toxicol.

